# Divergence, Convergence, and Therapeutic Implications: A Cell Biology Perspective of C9ORF72-ALS/FTD

**DOI:** 10.1186/s13024-020-00383-7

**Published:** 2020-06-08

**Authors:** Xiaoqiang Tang, Arturo Toro, Sahana T.G., Junli Gao, Jessica Chalk, Björn E. Oskarsson, Ke Zhang

**Affiliations:** 1 Department of Neuroscience, Mayo Clinic, Jacksonville, FL USA; 2 Department of Neurology, Mayo Clinic, Jacksonville, FL USA; 3 Neuroscience Graduate Program, Mayo Clinic Graduate School of Biomedical Sciences, Jacksonville, FL USA

**Keywords:** C9orf72, Amyotrophic lateral sclerosis, Frontotemporal dementia

## Abstract

Ever since a GGGGCC hexanucleotide repeat expansion mutation in *C9ORF72* was identified as the most common cause of familial amyotrophic lateral sclerosis (ALS) and frontotemporal dementia (FTD), three competing but nonexclusive hypotheses to explain how this mutation causes diseases have been proposed and are still under debate. Recent studies in the field have tried to understand how the repeat expansion disrupts cellular physiology, which has suggested interesting convergence of these hypotheses on downstream, functional defects in cells, such as nucleocytoplasmic transport disruption, membrane-less organelle defects, and DNA damage. These studies have not only provided an integrated view of the disease mechanism but also revealed novel cell biology implicated in neurodegeneration. Furthermore, some of the discoveries have given rise to new ideas for therapeutic development. Here, we review the research progress on cellular pathophysiology of C9ORF72-mediated ALS and FTD and its therapeutic implication. We suggest that the repeat expansion drives pathogenesis through a combination of downstream defects, of which some can be therapeutic targets.

## Background

Amyotrophic lateral sclerosis (ALS) and frontotemporal dementia (FTD) are two fatal neurodegenerative diseases with no curative treatment. ALS is a motor neuron degenerative disease with a lifetime risk of one in 347 men and one in 436 women, whereas FTD is the most common form of dementia for people under the age of 45 [1, 2]. ALS is characterized by degeneration of upper and lower motor neurons, causing muscle weakness and atrophy, whereas FTD is characterized by frontotemporal lobar degeneration, which can cause a heterogeneous group of clinical presentations, including progressive declines in cognition, behavior, and/or language. Based on the clinical presentations, FTD can be categorized into three subtypes: behavioral variant FTD, semantic dementia, and progressive non-fluent aphasia [3, 4].

Both ALS and FTD are associated with cellular deposition of protein inclusions. For ALS, a pathological hallmark observed in ~98% cases is the mislocalization and aggregation of TAR-DNA binding protein 43 (TDP-43). Other less frequently observed neuropathology includes the aggregation of superoxide dismutase 1 (SOD1), fused in sarcoma (FUS), and some heterogeneous nuclear ribonucleoproteins (hnRNPs) ([5–8];). For FTD, ~40% cases exhibit TDP-43 pathology, and another ~40% cases exhibit pathology of microtubule-associated protein tau. Other less frequently observed neuropathology includes FUS pathology and ubiquitin-positive aggregates with the protein components uncharacterized [8–10].

Despite their symptomatic differences, ALS and FTD share clinical, neuropathological, and genetic features and are part of a common spectrum. Indeed, ALS and FTD can occur in the same family, and many patients develop signs of both diseases. Furthermore, both ALS and FTD can be related to TDP-43 and FUS neuropathology [9]. In addition, mutations in several genes, including chromosome 9 open reading frame 72 (*C9ORF72*), have been identified to cause both ALS and FTD [11–15].

**Fig. 1:**
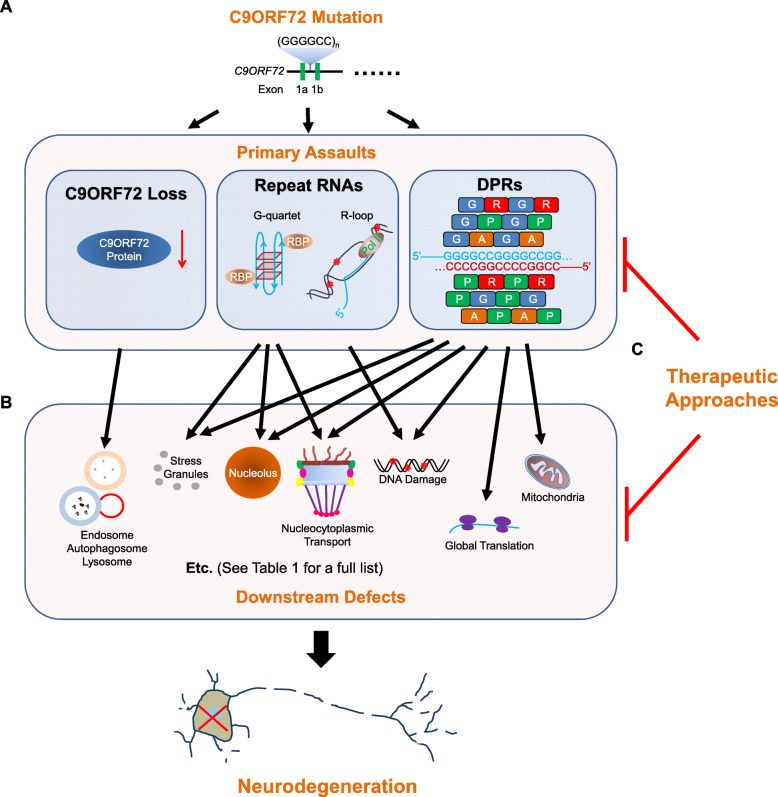
Summary of current cellular pathophysiological studies on C9ALS/FTD. **a** Three hypothesized primary assaults caused by the C9ORF72 mutation: 1) loss of C9ORF72 function, 2) repeat RNA forming either G-quartets or R-loops, toxic secondary structures that either sequester RBPs or cause DNA damage, respectively, and 3) DPRs. **b** The three primary assaults cause downstream, functional defects in nerve cells, and a combination of these defects causes neurodegeneration. **c** Therapeutic approaches can target either the primary assaults themselves, or their downstream effectors.

A GGGGCC (G_4_C_2_) hexanucleotide repeat expansion (HRE) in *C9ORF72* is the most common genetic cause of familial ALS (40%) and FTD (25%) and also presents in some sporadic cases (ALS: 8%; FTD:5%). The lengths of G_4_C_2_ HREs are greater than 30 in most patients but vary among individuals, with some patients carrying >1,000 repeats [12, 14]. How the G_4_C_2_ HRE causes neurodegeneration is not fully understood. Past studies have suggested that the toxicity arises from one or more of the following assaults (Figure 1A): 1) loss of C9ORF72 due to aborted transcription, 2) bi-directionally transcribed G_4_C_2_ and G_2_C_4_ repeat RNAs from the HREs [16, 17], and/or 3) dipeptide repeat proteins (DPRs) translated from the repeat RNAs, via repeat-associated, non-ATG (RAN) translation [18–22]. As the DPR translation is ATG-independent, it occurs in all three frames bi-directionally, leading to five different DPR species: poly-(glycine-alanine, or GA) and (glycine-arginine, or GR) from the sense (G_4_C_2_) transcript, poly-(proline-alanine, or PA) and (proline-arginine, or PR) from the antisense (G_2_C_4_) transcript, and poly-(glycine-proline, or GP) from both the sense and antisense transcripts.

Consistent with this idea, loss of C9ORF72 mRNA and proteins, G_4_C_2_, G_2_C_4_ repeat RNA foci, and aggregation of DPRs have been observed in patient tissues and model systems. Furthermore, some of these assaults can indeed cause neurodegeneration and/or are cytotoxic in certain model systems. However, other studies also suggest evidence against any of these three hypotheses. These studies, with a goal of resolving the debate on these three assaults, have been extensively reviewed by others [23–27].

Besides research efforts to resolve this debate, recent studies on *C9ORF72*-mediated ALS/FTD (C9ALS/FTD) have related the three assaults to downstream, functional defects in cells. These studies identified molecular and cellular events that are crucial for neurodegeneration implicated in C9ALS/FTD, suggesting novel therapeutic targets for the disease (Figure 1 B and C). Moreover, these findings have greatly improved our understanding of fundamental cell biology beyond the scope of neurodegenerative diseases. Here, we review recent studies on C9ALS/FTD from a cell biology perspective, with a focus on cellular pathophysiology and therapeutic implications*.*

## Main Text

### Model Systems to Study C9ALS/FTD Cellular Pathophysiology

To study the pathomechanism of C9ALS/FTD, many cellular and animal models have been used, including yeast, C. *elegans*, *Drosophila*, zebrafish, mouse, and neurons derived from patient induced pluripotent stem (iPS) cells (iPSNs). Using these model systems, research has identified critical cellular events in C9ALS/FTD pathogenesis. We will review the strengths and contributions of different model systems in C9ALS/FTD research.

#### Yeast and *Drosophila*

Yeast and *Drosophila* do not have a *C9ORF72* homolog. However, their short generation time and ease to handle make them powerful genetic tools to study the gain-of-toxicity mechanism. Many yeast or fly models of C9ALS/FTD have been established by ectopically expressing the G_4_C_2_ repeat RNA and/or DPRs, which causes cell death or neurodegeneration [12, 28–35]. Studies in these models have related the *C9ORF72* gain of toxicity to arginine-containing DPRs [29, 33, 34]. Furthermore, large-scale genetic screens in these models have identified crucial pathogenic events [28, 29, 32, 36, 37] and proteins involved in the production of the repeat RNAs or DPRs [30, 31, 38–40]. Importantly, these findings have been further verified in higher model organisms and patients, suggesting the power of yeast and *Drosophila* in studying the C9ALS/FTD disease mechanism.

#### Mouse

Mouse *C9orf72* is homologous to human *C9ORF72* and thus, its knockout (KO) can be used to study the loss-of-function mechanism. However, mouse *C9orf72* does not contain G_4_C_2_ repeats. Thus, one must ectopically express the repeat RNAs or DPRs in mice, as in yeast and *Drosophila*, to study the gain-of-toxicity mechanism. Several loss- or gain-of-function mouse models have been established for C9ALS/FTD [41–46], among which the models with adeno-associated-virus (AAV)-mediated repeat RNA or DPR expression have shown robust motor neuron loss and locomotion defects. Studies in these models have not only validated findings from yeast, *Drosophila*, and cultured cells [47–50] but also discovered novel disease pathophysiology [51].

#### IPSN

So far, translating drugs identified in animal models to ALS/FTD patients have gained little success [52–54], suggesting a gap between animal research and clinical studies. The recently developed iPS technology provides a potential solution to fill this gap [55, 56], as the iPSNs are derived from patients. Using iPSNs as models, studies on C9ALS/FTD have not only validated findings from animal models but also identified important pathophysiological events, such as ER stress [57]. Furthermore, these iPSNs provide a platform for genetic and drug screens [58, 59], which can be done in the future.

#### Other Model Systems

Besides yeast, fly, mouse and iPSN models, *C. elegans* and zebrafish models have also been established to study the C9ALS/FTD mechanism [60–65]. These studies have provided insights into both the loss- and gain-of-function mechanisms.

#### Using Multiple Model Systems

A major challenge in disease research is that all model systems have limitations. Thus, validation across model systems has been a powerful approach in studying human disease pathogenesis. Since non-vertebrate models are quick and easy to handle, whereas mouse and iPSN models are more disease-relevant, an efficient strategy to study disease mechanism is to first use non-vertebrate models to identify potential mechanisms and then, validate the findings in mammals and patient-derived iPSNs. This strategy ensures both the quickness and disease relevance and has been very successful in studying the C9ALS/FTD pathophysiology.

### C9ALS/FTD Mechanisms

#### Loss of function

Consistent with the loss-of-function hypothesis, *C9ORF72* transcript levels are decreased in some C9ALS/FTD patients [12, 66–70]. However, whether and how C9ORF72 loss contributes to pathogenesis is unclear. While *C9ORF72* knockdown causes motor neuron degeneration in *C. elegans* and zebrafish [60, 65], *C9ORF72* KO mice do not exhibit neurodegeneration or motor deficits [42, 45, 71–73], suggesting that C9ORF72 loss is insufficient to cause the disease in mammals. However, this does not exclude a contribution of C9ORF72 loss to pathogenesis. Indeed, recent studies in patient iPSNs or mice suggested that C9ORF72 loss can synergize with its gain of toxicity—*C9ORF72* KO enhances the sensitivity of iPSNs to toxic DPRs [74], and C9ORF72 loss exacerbates neurodegeneration caused by HRE gain of toxicity in mouse models [75]. The latter study also shows that C9ORF72 loss disrupts autophagy, accompanied by an increase in DPR levels [75], raising the possibility that C9ORF72 loss compromises the autophagolysosomal clearance of DPRs.

##### Cellular Vesicle Trafficking Impairment

C9ORF72 protein is structurally similar to Rab guanine nucleotide exchange factors (RabGEFs) and functions as a RanGEF when complexing with two other proteins, WDR41 and SMCR8 [76]. RabGEFs activate Rab proteins, a group of small GTPases that regulate membrane trafficking in cells. Consistent with these findings, C9ORF72 localizes to endosomes, autophagosomes, and lysosomes, where it colocalizes with several Rab proteins [77, 78]. Furthermore, siRNA against *C9ORF72* disrupts endocytosis and autophagy, leading to subcellular aggregation of p62 and/or TDP-43 in cultured neurons [77]. In agreement with these data, overexpressing C9ORF72 activates autophagy, as indicated by upregulated autophagosome formation. Interestingly, it is suggested that C9ORF72 activates autophagy via ULK1, a target protein of TBK1 [79, 80]. Since loss of TBK1 can also cause ALS and FTD [13], these data suggest that impaired autophagy may be a common pathogenic pathway in both C9ORF72- and TBK1-mediated ALS/FTD.

In agreement with the role of C9ORF72 in cellular vesicle trafficking, *C9ORF72* KO mice exhibit endolysosomal and/or autophagic defects, but these defects vary across different tissues. In macrophages, *C9ORF72* KO impairs autophagy and endolysosomal trafficking [45], whereas, in brain cells or fibroblasts, it increases autophagic flux [73]. In addition, *C9ORF72* KO also suppresses mTOR signaling and increases the nuclear level of TFEB, a master regulator of lysosomal biogenesis, in fibroblasts [73], suggesting that C9ORF72 regulates autophagy at multiple steps in different tissues.

In summary, C9ORF72 functions as a RabGEF when complexing with other proteins, which regulates vesicle trafficking in cells. Its loss causes endolysosomal and autophagic defects both *in vitro* and *in vivo*. While insufficient to initiate neurodegeneration, C9ORF72 loss impairs autophagy, which may contribute to neurodegeneration when combined with the gain-of-toxicity mechanism.

#### Gain of toxicity

While the G_4_C_2_ HRE leads to loss of C9ORF72, most evidence suggests that it causes diseases via a gain-of-toxicity mechanism. Indeed, the HRE produces G_4_C_2_ and C_4_G_2_ repeat mRNAs, which are believed to be responsible for the toxicity. However, how repeat mRNAs cause toxicity is unclear. So far, three models have been proposed: 1) The repeat mRNAs can form guanine-quadruplex (G-quartet) secondary structures, which bind to RNA-binding proteins (RBPs) and lead to their loss of function [16, 17, 37]; 2) The repeat mRNAs can form hybrids called “R-loops” with DNA double strands, which cause DNA damage [81]; 3) The repeat-containing unspliced mRNAs [82, 83] or spliced intronic RNA [84] can undergo RAN translation to generate DPRs, which are toxic [22, 29, 34, 85, 86].

These three models are nonexclusive, but the first two argue that the repeat mRNAs cause cytotoxicity by their secondary structure, whereas the third one argues that they cause cytotoxicity through DPRs. Consistent with these models, both sense and antisense repeat RNA foci, R-loops, and aggregates of all five DPR species have been observed in C9ALS/FTD patient tissues and model systems [12, 18, 20, 21, 48, 81, 87, 88]. In addition, antisense oligonucleotides (ASOs) or knockdown of transcription elongation factors reduces the levels of both repeat RNAs and DPRs and suppresses HRE-mediated toxicity in multiple C9ALS/FTD model systems [16, 31, 38, 40, 71]. However, whether the repeat RNAs cause cytotoxicity via their secondary structures or DPRs is under debate.

Small molecules targeting the RNA secondary structures have been shown to suppress cytotoxicity or neurodegeneration in C9ALS/FTD models, but these molecules also reduce DPR levels [37, 89, 90]. To distinguish the effects of the RNA secondary structures from DPRs, Mizielinska et al. [33] have generated *Drosophila* models expressing G_4_C_2_ repeats interrupted by stop codons in all frames (the “RNA-only,” or “RO,” flies) [33]. These flies express mRNAs with G-quartet secondary structures, but no DPR, and do not undergo neurodegeneration, whereas flies expressing regular G_4_C_2_ repeats exhibit severe neurodegeneration, suggesting that the G-quartet is non-toxic. This idea is supported by studies in another C9ALS/FTD *Drosophila* model, which exhibits RNA foci, but no detectable DPR or overt neurodegeneration [34]. However, it is unclear whether the G-quartets from the “RO” RNA bind to the same RBPs as the regular repeat RNA or whether the “RO” RNA forms R-loops. Importantly, the evidence against secondary-structure-mediated toxicity has so far been limited to studies in *Drosophila*, whereas in Zebrafish, both sense and antisense “RO” RNAs have been shown to exert neuronal toxicity [64]. To better understand the role of the secondary structures of the repeat RNAs in C9ALS/FTD pathogenesis, future studies can test the “RO” constructs in mammalian models.

Despite the controversy over whether the RNA secondary structures play a role in pathogenesis, it is generally agreed that DPRs are cytotoxic. Many studies have shown that DPRs encoded by alternative codons(i.e. non-HRE) cause toxicity in multiple model systems (reviewed in [24]). In addition, genetically or pharmacologically inhibiting DPR translation suppresses neuronal defects in C9ALS/FTD models, without affecting RNA foci [31, 33, 39, 91]. While the overall DPR pathology does not correlate with the affected brain regions in patients [88, 92–94], poly(GR) pathology does [95, 96]. Together, these studies suggest a critical role of DPRs in pathogenesis.

Among all five DPR species, the arginine-containing DPRs, i.e. poly(GR) and poly(PR), are the most toxic. In fly models, poly(GR) and poly(PR), but not other DPR species, cause neurodegeneration [33]. Consistent with these findings, poly(GR) and poly(PR) exhibit cytotoxicity in other model systems, including yeast, worm, mouse, and cultured cells, whereas neither poly(GP) nor poly(PA) is toxic in cultured cells [32, 49, 51, 62, 85, 97, 98]. For poly(GA), some studies reported its toxicity in mice and cultured neurons [50, 86, 99, 100]. However, others reported little or no poly(GA) toxicity in flies or cultured neurons, whereas poly(GR) and/or poly(PR) expressed in the same systems exhibit strong toxicity [29, 33, 98]. Thus, poly(GA) can be toxic under certain conditions but is much less toxic compared to poly(GR) and poly(PR), when these DPRs are expressed at similar levels. In agreement with these findings, mass spectrometry analyses have identified more than 200 endogenous proteins to specifically interact with poly(GR) and poly(PR) when these DPRs are overexpressed in cultured cells, whereas only six proteins specifically interact with poly(GA), and none specifically interact with poly(GP) and poly(PA) [36].

Although the repeat RNAs and some DPR species cause little or no toxicity when expressed alone, it is possible that they can enhance poly(GR)- or poly(PR)-mediated cytotoxicity. For example, some RNA has been shown to promote liquid-liquid phase separation (LLPS) of poly(GR) and poly(PR), a process associated with poly(GR)- and poly(PR)-mediated cytotoxicity [101]. Furthermore, poly(GA) and poly(GP) sometimes co-aggregate with poly(GR) and/or poly(PR), suggesting that they may enhance the aggregation propensity of poly(GR) and poly(PR) [86].

**Fig. 2:**
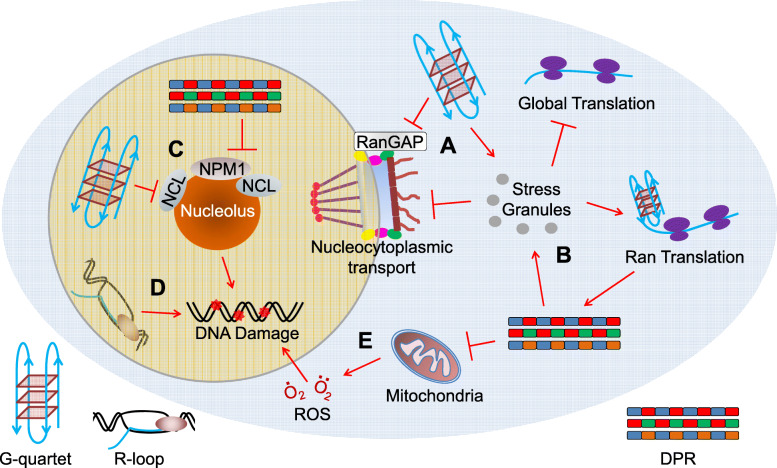
Connections among functional defects downstream of repeat RNAs and DPRs. **a** G-quartets formed by repeat RNAs disrupt nucleocytoplasmic transport by sequestering RanGAP. It also promotes stress granule assembly. **b** DPRs induce stress granule assembly, which disrupts nucleocytoplasmic transport, inhibits global translation, and enhances RAN translation. Enhanced RAN translation produces more DPRs, forming a feedforward loop. **c** G-quartets formed by repeat RNAs disrupt nucleoli by sequestering NCL, whereas DPRs disrupt nucleoli by co-phase separating with NPM1 and NCL. In addition, DPRs cause DNA damage via NPM1 and nucleolar defects. **d** Repeat RNAs form R-loops, which causes DNA damage. **e** Cytoplasmic DPRs disrupt mitochondrial function, producing ROS that causes DNA damage. These discoveries not only provide us with an integrated view of C9ALS/FTD pathogenic mechanism but also help us better understand the fundamental biology underlying neurodegeneration.

Despite the debate over the RNA-secondary-structure- versus DPR-mediated toxicities, recent studies have suggested that they converge on the same downstream, functional defects in cells, including nucleocytoplasmic transport disruption, membrane-less organelle defects, and DNA damage (Figure 2). Indeed, emerging evidence has suggested the importance of these defects in pathogenesis, which will be the focus of this review. In addition, we will also discuss other functional defects implicated in C9ALS/FTD.

##### Nucleocytoplasmic Transport Disruption

Several studies have independently identified a critical role of nucleocytoplasmic transport disruption in C9ALS/FTD, which can stem from either RNA secondary structure or DPRs [28, 29, 32, 37, 102]. One study from us has shown that the G-quartets formed by the sense repeat RNA bind to and sequester RanGAP1, a key regulator of nucleocytoplasmic transport, which impairs the nuclear import of proteins in C9ALF/FTD fly and iPSN models [37]. Importantly, a nuclear export inhibitor, KPT-276, restores the balance between import and export and suppresses neurodegeneration in the fly model, suggesting nucleocytoplasmic transport defects as a potential therapeutic target. In parallel to this study, another study has shown that the sense repeat RNA and/or arginine-containing DPRs disrupt the nuclear export of mRNAs in another C9ALS/FTD fly model [29]. Consistent with these studies, genetic screens have implicated nucleocytoplasmic transport disruption in yeast or flies expressing poly(PR) or C9ALS/FTD patient iPSNs [28, 32]. The importance of nucleocytoplasmic transport disruption is further supported by immunohistochemistry staining in C9ALS/FTD patient or mouse tissues, which exhibit aggregation of nucleocytoplasmic transport factors [37, 47, 50, 51].

The idea that the G-quartets disrupt nucleocytoplasmic transport by binding to RanGAP1 [37] is further supported by a large-scale interactome study identifying RanGAP1 as an interactor of GC-enriched RNAs [103]. However, how DPRs disrupt nucleocytoplasmic transport is under debate. Shi et al. [102] have shown that a chemically synthesized poly(PR) peptide blocks nuclear pore by binding to nucleoporins, leading to nucleocytoplasmic transport defects [102]. In contrast, a recent study has failed to detect similar defects induced by the same peptide [104], possibly due to different experimental settings in these two studies. The second study challenges the idea that DPRs directly block nuclear pores, raising the possibility that DPRs disrupt nucleocytoplasmic transport through an indirect mechanism.

The discovery of DPRs disrupting nucleocytoplasmic transport has triggered a surge of further studies, which have identified nucleocytoplasmic transport disruption as a common cellular defect in protein-misfolding diseases [105–108]. Upon protein misfolding stress, cells halt their translation by embedding their mRNAs in large RNA/protein condensates called stress granules, in which many nucleocytoplasmic transport factors are sequestered. As DPRs have been shown to induce stress granule assembly in some cells, it is likely that DPRs disrupt nucleocytoplasmic transport by sequestering transport factors in stress granules. In agreement with this hypothesis, we have shown that many transport factors localize to DPR-induced stress granules and stress granule inhibitors partially suppress nucleocytoplasmic transport defects in cells overexpressing DPRs [109]. Furthermore, Vanestte et al. (2019) have observed a correlation between the presence of stress granules and nucleocytoplasmic transport defects in cells expressing DPRs at low levels [104]. Together, these findings suggest that DPRs can disrupt nucleocytoplasmic transport through stress granule assembly.

##### Membrane-less Organelle Defects

Stress granules are a group of membrane-less organelles, subcellular compartments that lack surrounding lipid membranes and perform certain biochemical functions. These organelles are protein/RNA condensates enriched in proteins with low-complexity domains (LCDs). Many LCD-containing proteins are aggregate-prone and can undergo LLPS, a process in which molecules demix in an aqueous environment. It has been shown that proper LLPS plays a key role in the assembly, dynamics, and functions of membrane-less organelles, whereas aberrant LLPS can cause aggregation of LCD-containing proteins, as well as defects in these organelles (reviewed in [110]).

In C9ALS/FTD, both the sense repeat RNA and DPRs have been shown to disrupt membrane-less organelles, including stress granules and nucleoli. Indeed, poly(GR) and poly(PR) interactomes are enriched in protein components of stress granules, nucleoli, and other membrane-less organelles, and many of these proteins also genetically interact with poly(GR) or poly(PR) [36, 111]. Furthermore, *in vivo* studies have suggested an adverse effect of poly(PR) on heterochromatin protein complexes [51], another form of membrane-less organelles. We will discuss these studies in this section.

- Stress Granule

Stress granules are cytoplasmic RNA/RBP condensates, but certain stimuli can convert them into protein aggregates. Interestingly, several ALS/FTD proteins, including TDP-43, FUS, Ataxin-2, TIA1, and hnRNPs, are stress granule proteins. Since the cytoplasmic aggregation of TDP-43, FUS, and hnRNPs is a pathological hallmark of ALS/FTD, including C9ALS/FTD, stress granules are believed the key to ALS/FTD pathogenesis. So far, this idea has been supported by genetic, cell biology, and pathological evidence in many model systems or patients (reviewed in [7]). However, other studies have shown that TDP-43 can aggregate in cultured cells via stress-granule-independent routes [112, 113]. Furthermore, some TDP-43 aggregates in ALS/FTD model systems or patients do not colocalize with stress granule markers [114, 115], suggesting that stress granules are insufficient to cause all TDP-43 pathology in ALS/FTD. Besides its role in triggering protein aggregation, stress granule assembly inhibits global translation and disrupts nucleocytoplasmic transport [82, 84, 109, 116], which can contribute to neurodegeneration. Thus, stress granules contribute to ALS/FTD pathogenesis through multiple mechanisms.

In C9ALS/FTD, both the G_4_C_2_ repeat RNA and DPRs promote stress granule assembly. The G-quartets formed by the G_4_C_2_ repeat RNA bind to and condense RBPs that are stress granule components, promoting RBP LLPS. Consistent with these findings, cells transfected with synthetic G_4_C_2_ repeat RNA exhibit stress granules, which occasionally colocalize with the repeat RNAs [117]. However, it is unclear whether these stress granules are caused by G-quartets or DPRs. Indeed, all five DPRs have been shown to induce stress granule assembly without additional stress [36, 98, 101, 109, 118]. Importantly, poly(GR) and poly(PR) localize to stress granules that they induce, bind to, alter the LLPS propensity, and impair the dynamics of stress granule proteins [36, 101]. As the dynamics of proteins negatively correlates with their propensity to aggregate, these studies suggest that poly(GR) and poly(PR) promote the aggregation of stress granule proteins. Indeed, poly(GR) co-aggregates with several stress granule proteins in the brain of C9ALS/FTD patients and mouse models [47, 49], suggesting that poly(GR) causes aggregation of stress granule proteins *in vivo*. Consistent with the role of stress granules in translation repression, poly(GR) and poly(PR) have been shown to suppress global translation in multiple model systems [36, 49, 104, 119, 120].

In accord with these mechanistic studies, C9ALS/FTD iPSNs exhibit an increased propensity to form, and a decreased rate to clear, stress granules [121]. Importantly, inhibiting stress granule assembly by either knockdown of genes or chemical inhibitors suppresses neurodegeneration in C9ALS/FTD iPSN and fly models [109], suggesting the potential of stress granules as a therapeutic target.

b) Nucleolus

Nucleoli are nuclear RNA/RBP condensates where ribosomal biogenesis occurs. They contain many proteins that undergo LLPS, such as NCL and NPM1. Both G_4_C_2_ repeat RNA and arginine-containing DPRs have been shown to disrupt nucleolar structure and function. Haeusler et al. [17] have shown that the G-quartets formed by G_4_C_2_ repeat RNA bind to NCL, causing NCL mislocalization and nucleolar stress [17]. Consistent with these data, nucleoli are expanded in cultured lymphocytes from C9ALS/FTD patients, and the nucleolar function is impaired in patient motor cortices. Together, these findings suggest that repeat RNAs disrupt nucleolar structure and function. While Haeusler et al. [17] reported expanded nucleoli in C9ALS/FTD lymphocytes, another study has observed no nucleolar size change in postmortem brains [88]. However, the results from the latter study could be due to pathological heterogeneity in brain cells. Indeed, a third study has shown that the nucleolar size is increased in C9ALS/FTD postmortem brains if only cells with repeat RNA foci are quantified [122], suggesting that the repeat RNAs indeed increase the nucleolar size. Interestingly, this study has also shown that brain cells without poly(GR) aggregation exhibit reduced nucleolar size, whereas cells containing poly(GR) aggregation exhibit enlarged nucleoli [122], suggesting a role of poly(GR) in nucleolar stress.

Several studies have shown that either overexpressed or chemically synthesized poly(GR) and poly(PR) localize to nucleoli and disrupt nucleolar functions [36, 85, 98, 101, 118, 123]. Furthermore, poly(GR) and poly(PR) undergo LLPS and bind to many nucleolar proteins, including NCL and NPM1, which disturbs LLPS and impair the dynamics and function of NCL and NPM1 [36, 101, 111, 123]. Together, these data suggest that poly(GR) and poly(PR) disrupt nucleolar function in cultured cells by impairing LLPS of key nucleolar proteins.

Despite their nucleolar toxicity in cultured cells, poly(GR) and poly(PR) rarely localize to nucleoli in C9ALS/FTD patient and mouse models and fail to induce nucleolar stress when overexpressed in mouse brains [49, 51, 88]. To define the precise role of poly(GR) and poly(PR) in nucleolar function *in vivo*, future studies may focus on whether they disrupt nucleoli when combined with the repeat RNAs and/or other DPRs in mouse models.

c) Heterochromatin Protein Complex

In a C9ALS/FTD mouse model overexpressing poly(PR), Zhang et al. [51] have unexpectedly discovered that poly(PR) rarely localizes to nucleoli [51], despite previous findings in cultured cells. Interestingly, poly(PR) mostly localizes to heterochromatin, DNA/protein condensates containing transcriptionally silent chromatin, in these mice. The authors have also discovered that poly(PR) localizes to heterochromatin in some C9ALS/FTD patient neurons. Consistent with these findings, transcripts from heterochromatin accumulate in both these mice and patients, suggesting heterochromatin defects.

Heterochromatin formation is mediated by heterochromatin protein 1 (HP1) proteins, which bind to chromatin and recruit other chromatin-interacting proteins. Recent studies have suggested that the heterochromatin compartment is a membrane-less organelle formed by LLPS of HP1α, an HP1 protein that contains LCDs [124, 125]. Interestingly, poly(PR) disrupts HP1α LLPS and reduces HP1α levels in mice overexpressing poly(PR) [51], suggesting a mechanism by which poly(PR) disrupts heterochromatin. These findings and previous findings in cultured cells [36, 101, 111] have converged on the adverse effects of poly(PR) on LLPS of LCD-containing proteins.

d) Other Membrane-less Organelles

In addition to stress granule and nucleolar proteins, components of other membrane-less organelles, including nuclear speckles and Cajal bodies, have been identified as physical and/or genetic interactors of poly(GR) and/or poly(PR) [36, 111]. Furthermore, these DPRs also alter nuclear speckle dynamics and Cajal body assembly in cultured cells, possibly through similar mechanisms as they disrupt stress granules and nucleoli.

In summary, both the G_4_C_2_ repeat RNA and DPRs, especially poly(GR) and poly(PR), disrupt multiple types of membrane-less organelles both *in vivo* and *in vitro* by binding to and impairing LLPS of proteins in these organelles. Notably, both the G_4_C_2_ repeat RNA and arginine-containing DPRs have been shown to undergo LLPS, but whether their LLPS propensity correlates with their toxicity on membrane-less organelles is unclear. Nevertheless, it has been shown that poly(GR) co-aggregates with stress granule proteins in both C9ALS/FTD mouse and patient neurons [51, 109]. Thus, it is intriguing to hypothesize that LLPS triggers the aggregation of repeat RNAs, arginine-containing DPRs, and their interactors in membrane-less organelles.

##### DNA Damage

Besides impaired nucleocytoplasmic transport and membrane-less organelles, DNA damage can also be caused by either repeat RNAs or DPRs and has been observed in multiple model systems of C9ALS/FTD, as well as in patients [81, 97, 126, 127]. So far, studies have identified several routes by which the repeat RNAs or DPRs can cause DNA damage. Firstly, the repeat RNAs cause DNA double-strand breaks via R-loops. Secondly, the repeat RNAs and poly(GA) cause DNA damage by disrupting the ataxia-telangiectasia-mutated-signaling pathway, a critical component in DNA damage repair, and/or reducing the level of hnRNP A3 [81, 127]. Thirdly, poly(GR) interacts with mitochondrial proteins and disrupts mitochondrial function, leading to increased oxidative stress and DNA damage in iPSNs [126]. As DNA damage in these iPSNs can be partially suppressed by antioxidants, it is suggested that poly(GR) causes DNA damage partially through oxidative stress [126, 128]. Fourthly, poly(GR) and poly(PR) may cause DNA damage by disrupting the function of NPM1, a nucleolar protein that functions in DNA damage repair [97]. These mechanisms are non-exclusive and likely co-exist in patients. Notably, several proteins involved in DNA damage repair, e.g. NPM1, TDP-43, and FUS, undergo LLPS and are known to interact with poly(GR) and poly(PR). Thus, future studies may test the possibility that poly(GR) and poly(PR) damage DNA by disrupting LLPS of these proteins.

Although DNA damage is generally believed to cause neurodegeneration, direct evidence is lacking to demonstrate that it contributes to C9ALS/FTD pathogenesis. Resolving R-loops by Senataxin or suppressing oxidative stress by SOD1 and/or catalase ameliorates repeat-RNA- and/or DPR-mediated toxicity [81, 126]. However, these approaches target factors upstream of DNA damage. Given the technical challenges to modulate DNA damage directly, future studies can focus on up- and downstream factors of DNA damage.

##### Other Functional Defects

In addition to nucleocytoplasmic transport disruption, membrane-less organelle defects, and DNA damage, many other functional defects have been implicated in C9ALS/FTD. For example, poly(GR) has been shown to disrupt mitochondrial function by sequestering key mitochondrial proteins in iPSN and/or mouse models [126, 128]. Consistent with these findings, mitochondrial morphology is disrupted in C9ALS/FTD fibroblasts [129]. Poly(GR) and poly(PR) have also been shown to disrupt axonal transport of mitochondria and vesicles [130]. Furthermore, poly(GA) has been shown to impair the proteasome system [50, 131]. In addition, dysregulation of RNA editing has been implicated in C9ALS/FTD [132]. For a list of functional defects implicated in C9ALS/FTD, please see Table 1. These findings suggest the complex pathophysiology of C9ALS/FTD, with a remaining question as to how these defects connect to each other.

**Table 1:** Cellular pathophysiological processes implicated in C9ALS/FTD

Cause	Affected Cellular Organelles or Processes	Implicated in Model systems						Patient Tissue
		Yeast	Worm	Drosophila	Mouse	Cultured Non-patient Cells	Patient iPSNs	
Loss of C9ORF72	Endolysosome		Yes		Yes	Yes	Yes	
	Autolysosome		Yes		Yes	Yes		
Repeat RNA and DPR	Nucleocytoplasmic transport	Yes		Yes	Yes	Yes	Yes	Yes
	Nucleolus			Yes	Yes	Yes	Yes	Yes
	RNA granules				Yes*	Yes		Yes^a^
	DNA damage				Yes	Yes	Yes	
DPR	Heterochromatin				Yes			Yes
	Mitochondria				Yes	Yes	Yes	
	Transcription and/or translation	Yes		Yes	Yes	Yes	Yes	Yes
	Proteasome				Yes	Yes		
	Reactive oxygen species					Yes	Yes	
	Axonal transport						Yes	
Undefined	ER stress				Yes		Yes	
	RNA editing						Yes	
	Excitotoxicity						Yes	Yes
	Cytoskeleton				Yes		Yes	
	Glia-related				Yes			Yes

^a^Aggregation of proteins that are components of RNA granules were observed.

##### Connections among Functional Defects

Eukaryotic cells, including neurons, are highly ordered, dynamic entities with intricate organization. Defects in one cellular organelle or process usually disrupt others. Indeed, many cellular functional defects in C9ALS/FTD are connected. Stress granule assembly has been shown to disrupt nucleocytoplasmic transport in C9ALS/FTD by sequestering transport factors in stress granules [109], whereas disrupted nucleocytoplasmic transport may cause RNA editing enzyme ADAR2 to mislocalize to the cytoplasm, which causes RNA editing defects in C9ALS/FTD [132]. In addition, mitochondrial defects have been shown to cause DNA damage in C9ALS/FTD via reactive oxygen species [126], whereas nucleolar defects may also contribute to DNA damage in C9ALS/FTD via disrupted NPM1 function [97]. Furthermore, DPR-induced stress granule assembly inhibits global translation but selectively enhances RAN translation, thereby promoting DPR production [82, 84, 116]. These findings have not only revealed molecular cascades and feedforward mechanisms underlying the pathogenesis but also help us better understand the intricate organization of eukaryotic cells—a fundamental issue in cell biology.

### Therapeutic Development

Since the major cause of C9ALS/FTD appears to be the gain of toxicity derived from the repeat RNAs and their translational products, DPRs, methods to eliminate these toxic species have been actively explored. Particularly, an ASO therapy is more advanced than other therapies in clinical development. Notably, the repeat RNAs and DPRs are toxic because they disrupt certain function(s) of cells. Thus, targeting functional defects downstream of the repeat RNAs and/or DPRs holds great therapeutic potential. Indeed, a chemical compound targeting nucleocytoplasmic transport, which stems from our discoveries [37], is currently in an ALS clinical trial. In this section, we will review the current progress on C9ALS/FTD therapeutic development and suggest potential ideas for future studies.

#### Targeting repeat RNAs and/or DPRs

##### *C9ORF72* ASO

ASOs are synthetic single-stranded oligonucleotides that can bind to their target RNAs with very high specificity. This binding can restore, modify, inactivate, or promote the degradation of the RNAs. ASOs are stable and effective in the central nervous system, and an ASO modifying gene expression has been used to successfully treat spinal muscular atrophy, another motor neuron degenerative disease [133–136]. In addition, an ASO that activates RNase-H-mediated mRNA degradation has exhibited promising effects on patients with SOD1-related ALS (ALS1) and is now in a phase III study (https://clinicaltrials.gov/ct2/show/NCT02623699?term=Tofersen&draw=2&rank=1), supporting the efficacy of ASOs in treating ALS caused by a gain-of-toxicity mutation.

For C9ALS/FTD, current ASO therapies focus on degrading the sense transcripts. The ASOs against *C9ORF72* have been shown to suppress pathological and pathophysiological defects, as well as neurodegeneration, in multiple model systems [16, 37, 71, 137–139]. Phase I clinical studies with an ASO that activate RNase-H-mediated degradation of the sense transcripts are underway through Ionis and Biogen (https://clinicaltrials.gov/ct2/show/NCT03626012).

Despite its promise, there are concerns related to ASO therapy. First, the current ASO used in clinical studies does not directly impact the antisense transcript, which may leave part of the pathology unaffected. Furthermore, SMA and ALS1 mainly affect lower motor neurons, whereas C9ALS/FTD is often related to strong upper-motor-neuron affliction. To treat C9ALS/FTD, the pharmacokinetics and pharmacodynamics of ASOs in upper motor neurons must be determined. It is also worth noting that ASOs treating SMA and ALS have different effects on mRNAs—the ASO for SMA blocks an internal splicing site whereas the ASOs for ALS1 and C9ALS/FTD degrade mRNAs. Thus, the clinical effects of these ASOs may be different.

##### Other Molecules

Small molecules modulating G_4_C_2_ RNA G-quartets or antibodies against poly(GA) have been shown to suppress HRE-mediated toxicity in cell and/or *Drosophila* models of C9ALS/FTD [37, 90, 140]. Future studies may focus on their effects in mammals. Furthermore, Metformin, an FDA-approved drug to treat diabetes, has been shown to reduce RAN translation. An ongoing clinical study is assessing its safety and tolerability in C9ALS/FTD patients (https://clinicaltrials.gov/ct2/show/NCT04220021).

In addition to the ASOs and molecules discussed above, proteins implicated in the production of repeat RNAs or DPRs [30, 38, 91] may also be therapeutic targets. Furthermore, AAV-mediated delivery of gene-silencing tools, such as CRISPR/Cas9, is in its infancy. Future research can test these approaches.

#### Targeting Downstream Defects

##### Nuclear Export Inhibitor KPT-350 (BIIB100)

A pathological hallmark of ALS/FTD, including C9ALS/FTD, is cytoplasmic mislocalization of TDP-43 [8, 9], potentially due to impaired nuclear import. Thus, inhibiting nuclear export may restore the nuclear import/export balance and suppress neurodegeneration. Indeed, we have shown that an Exportin-1 inhibitor, KPT-276, suppresses nucleocytoplasmic transport defects, as well as neurodegeneration, in a C9ALS/FTD fly model [37]. In addition, another Exportin-1 inhibitor, KPT-350, a.k.a. BIIB100, suppresses neurodegeneration in a rat model of TDP-43-mediated ALS/FTD [141], suggesting its therapeutic potential. Currently, a phase I clinical study on KPT-350 is underway (https://clinicaltrials.gov/ct2/show/NCT03945279). However, recent studies have suggested that the nuclear export of TDP-43 is not mediated by Exportin-1 [142, 143] and KPT-350 does not suppress TDP-43 mislocalization in the ALS/FTD rat model [141], raising the question as to how KPT-350 suppresses neurodegeneration. Interestingly, KPT-350 has been shown to suppress inflammatory responses in the central nervous system [144], possibly because Exportin-1 exports transcription factors implicated in anti-inflammatory responses from the nucleus. Hence, it is possible that inflammatory responses contribute to C9ALS/FTD pathogenesis.

##### Stress Granule Inhibitors and Ataxin-2 ASO

Stress granule assembly is believed to contribute to ALS/FTD pathogenesis by triggering the aggregation of TDP-43, FUS, and other hnRNPs. Consistent with this hypothesis, inhibiting stress granule assembly using chemical inhibitors or ASOs against Ataxin-2, an essential stress granule component, has been shown to suppress defects in iPSN and/or *Drosophila* models of C9ALS/FTD [109]. Stress granule inhibitor 2BAct has also been shown neuroprotective in a mouse model of Vanishing White Matter, another neurodegenerative disease [145]. In addition, Ataxin-2 ASOs have been shown to suppress neurodegeneration in mouse models of TDP-43-related ALS or spinocerebellar ataxia 2 [146, 147]. Together, these findings have suggested the therapeutic potential of these approaches. Recently, a high-content drug screen has identified ~100 small molecules that modulate stress granules in cultured cells [148], providing a large pool of candidate drugs for future *in vivo* studies.

In addition to nucleocytoplasmic transport and stress granules, other downstream, functional defects may also be therapeutic targets. Importantly, given that some of these defects also present in other types of ALS or FTD, strategies targeting these defects are likely able to be translated from C9ALS/FTD to other ALS/FTD cases. For example, the clinical trial on KPT-350 also includes sporadic ALS patients. However, before targeting a downstream defect, one must carefully evaluate whether this defect is indeed the cause, rather than simply a consequence or byproduct, of neurodegeneration. Thus, rescuing with genetic and pharmacological manipulations in model systems is highly recommended before further analyses.

## Conclusions

Like many other neurodegenerative diseases, C9ALS/FTD has a complex pathophysiology. Although it is widely accepted that the G_4_C_2_ HRE causes neurodegeneration via a gain of toxicity, emerging evidence suggests that loss of C9ORF72 also plays an auxiliary role. Furthermore, both the loss and gain of function cause defects in many subcellular organelles/processes that contribute to C9ALS/FTD neurodegeneration. Importantly, some of these defects also occur in other forms of ALS/FTD and other neurodegenerative diseases (e.g. Alzheimer’s disease), as well as during cellular responses to a variety of stress. Thus, these studies also help us better understand not only the mechanisms of other diseases but also the principles of fundamental cell biology.

Despite recent progress, several questions remain unanswered. Firstly, some studies suggest the involvement of Golgi and the extracellular matrix in pathogenesis with the underlying mechanism unclear. Secondly, how glia contributes to the diseases is ill-defined. Importantly, it remains unclear whether the downstream defects caused by G_4_C_2_ HRE occur in parallel or in series. Identifying defects that occur early in pathogenesis is crucial, as targeting these events will likely yield clinical effects.

If the ASO approach will treat C9ALS/FTD in the future, as it does for SMA, one may question the value of studying downstream cellular defects. However, many of these defects also occur in sporadic ALS or FTD cases, which lack an obvious genetic cause. Thus, even from a clinical perspective, understanding the cellular and molecular basis of these diseases is still vital.

## Data Availability

Not applicable.

## Abbreviations

ALS: Amyotrophic lateral sclerosis
FTD: Frontotemporal dementia
TDP-43: TAR-DNA binding protein 43
FUS: Fused in sarcoma
HnRNP: Heterogeneous nuclear ribonucleoproteins
C9ORF72: Chromosome 9 open reading frame 72
G_4_C_2_: GGGGCC
HRE: Hexanucleotide repeat expansion
RAN: Repeat-associated, non-ATG
DPR: Dipeptide repeat protein
C9ALS/FTD: *C9ORF72*-mediated ALS and FTD
iPSN: Neurons derived from induced pluripotent stem cells
AAV: Adeno-associated-virus
LLPS: Liquid-liquid phase separation
LCD: Low-complexity domain
RBP: RNA-binding protein
ASO: Antisense oligonucleotide
