# Correction to: Divergence, Convergence, and Therapeutic Implications: A Cell Biology Perspective of C9ORF72-ALS/FTD

**DOI:** 10.1186/s13024-020-00390-8

**Published:** 2020-07-01

**Authors:** Xiaoqiang Tang, Arturo Toro, T. G. Sahana, Junli Gao, Jessica Chalk, Björn Oskarsson, Ke Zhang

**Affiliations:** 1 Department of Neuroscience, Mayo Clinic, Jacksonville, FL USA; 2 Department of Neurology, Mayo Clinic, Jacksonville, FL USA; 3 Neuroscience Graduate Program, Mayo Clinic Graduate School of Biomedical Sciences, Jacksonville, FL USA

**Correction to: Mol Neurodegeneration 15, 34 (2020)**

**https://doi.org/10.1186/s13024-020-00383-7**

*The original article* [1] *incorrectly displays 6th author, Björn Oskarsson’s name with middle initial. The correct presentation can be viewed in this Correction article.*
